# Primary care provider perceptions and experiences of implementing hepatitis C virus birth cohort testing: a qualitative formative evaluation

**DOI:** 10.1186/s12913-019-4043-z

**Published:** 2019-04-23

**Authors:** Vera Yakovchenko, Rendelle E. Bolton, Mari-Lynn Drainoni, Allen L. Gifford

**Affiliations:** 1 Center for Healthcare Organization and Implementation Research, Edith Nourse Rogers Memorial Veterans Hospital, 200 Springs Road (152), Building 70, Bedford, MA 01730 USA; 2 Brandeis University, Heller School for Social Policy and Management, Waltham, MA USA; 3 Center for Healthcare Organization and Implementation Research, VA Boston Healthcare System, Boston, MA USA; 4 Department of Health Law, Policy and Management, Boston University School of Public Health, Boston, MA USA; 5 Section of Infectious Diseases, Department of Medicine, Boston University School of Medicine and Public Health, Boston, MA USA

**Keywords:** Hepatitis C, Infectious diseases, Screening, Testing, Guidelines, Primary care, Veterans health

## Abstract

**Background:**

In 2014, the Department of Veterans Affairs (VA) adopted a screening test policy for hepatitis C virus (HCV) in all “Baby Boomers” - those born between 1945 and 1965. About 1 in 12 Veterans were estimated to be infected with HCV yet approximately 34% of the birth cohort remained untested. Early HCV diagnosis and successful antiviral treatment decrease the risk of onward transmission, cirrhosis, hepatocellular carcinoma, liver transplant, and death. Implementing evidence-based HCV screening in primary care has great potential to reduce morbidity and mortality. To inform design and implementation of a quality improvement intervention, we studied primary care provider (PCP) perceptions of and experiences with HCV birth cohort testing.

**Methods:**

We conducted a formative evaluation using qualitative semi-structured interviews guided by the integrated Promoting Action on Research Implementation in Health Services (i-PARIHS) framework. Twenty-two PCPs in six states across a large integrated US healthcare system were interviewed. Content analysis with a priori and emergent codes was performed on verbatim interview transcripts.

**Results:**

We identified three themes related to primary care provider HCV testing and linkage practices, as mapped to i-PARIHS constructs: 1) evaluating cues to HCV testing (innovation/evidence), 2) framing HCV testing decisions (recipients), and 3) HCV testing and linkage to care in the new treatment era (context). The most frequently reported HCV testing cue was an electronic clinical reminder alert, followed by clinical markers and the presence of behavioral risk factors. Most PCPs saw testing as routine, but less urgent, leading to some reluctance. Providers largely saw themselves as performing guideline-concordant testing, yet no performance data were available to assess performance. Given the recent availability of new HCV medications, many PCPs were highly motivated to test and link patients to specialty care for treatment.

**Conclusions:**

Our results suggest a multi-component intervention around awareness and education, feedback of performance data, clinical reminder updates, and leadership support, would address both a significant need, and be deemed acceptable and feasible to primary care providers.

**Electronic supplementary material:**

The online version of this article (10.1186/s12913-019-4043-z) contains supplementary material, which is available to authorized users.

## Background

Hepatitis C virus (HCV) is the most common blood borne infection in the United States, 3.5 million persons estimated to be chronically infected, 75% of whom were born between 1945 and 1965 [1]. Veterans are disproportionately affected—in 2014, an estimated nearly 9% of birth cohort Veterans in Department of Veterans Affairs (VA) care were HCV infected, yet 34% had never been HCV tested [2]. To improve detection of HCV, VA adopted birth cohort HCV testing in 2014.

Early detection through implementing evidence-based HCV testing has great potential to prevent onward transmission, reduce morbidity and mortality [3]. With the advent of curative interferon-free antiviral HCV medications, the importance of HCV detection has only intensified [4]. However, integration of guidelines into primary care practices is a non-trivial matter for already overburdened primary care providers (PCPs). Various quality improvement techniques have been proven effective, yet formative evaluations to understand barriers and facilitators are underutilized, thus strategy selection processes are often not systematic, altogether missing, or are insufficiently justified [5, 6].

The integrated Promoting Action on Research Implementation in Health Services (i-PARIHS) framework identifies implementation as a function of the innovation and its evidence base, individual and collective recipient characteristics, the local and broader organizational context, and how uptake is activated through the use of facilitation implementation strategies. i-PARIHS is well-suited as a guide for formative evaluation [7, 8]. At present, there is a paucity of research on the empirical application of i-PARIHS, and no qualitative studies on HCV screening practices in the new HCV treatment era.

The VA is the largest integrated healthcare system in the US, serving the world’s largest population of individuals with HCV. VA is an ideal environment to explore policy and implementation related to HCV testing, given its size, geographically dispersed hospitals and clinics, heterogeneity of sites and providers, and centralized policy structure with locally tailored implementation. To drive successful design and implementation of a quality improvement intervention, we sought to understand primary care provider (PCP) perceptions of and experiences with HCV birth cohort testing. A formative evaluation was undertaken to describe HCV screening, linkage to specialty care practices, and implementation barriers and facilitators among VA primary care providers after introduction of the birth cohort testing guidelines. The i-PARIHS framework was used as a lens through which to examine the data and inform design of an implementation study.

## Methods

### Design

We conducted a qualitative study employing semi-structured interviews with 22 primary care providers working with US Veterans across eight healthcare systems in New England. The Institutional Review Board for the Edith Nourse Rogers Memorial Veterans Hospital approved this study and informed consent was obtained from each participant.

### Participants

Participants were identified through a VA provider database and recruited through emails inviting them to participate in a one-time interview about their HCV testing practices. We used a stratified purposeful technique to sample PCPs in six states, seeking representation by provider type (physician, physician assistant, nurse practitioner), setting (hospital, community clinic), geography (rural, urban), and percent of full-time worked. We intentionally oversampled providers with larger panels to better represent typical care delivered.

### Data collection

We developed a semi-structured interview guide informed by PARIHS (precursor to the i-PARIHS framework). We inquired about PCPs beliefs about birth cohort testing, role of testing guidelines in decision-making, influences on decisions to offer testing, levels of priority of HCV testing relative to other clinical responsibilities, barriers and facilitators to testing, and linkage to specialty care practices (Additional file 1). Telephone interviews were conducted between September and November 2014, lasted up to an hour and averaged 20 min in length. They were audio-recorded and transcribed verbatim. We continued to enroll participants in the study until a cross-section of perspectives, and thematic saturation were achieved [9].

### Data analysis

We conducted content analysis using both a priori codes developed from interview guide questions, and emergent codes developed inductively from the data [10]. Two members (V.Y. and R.B.) of the study team independently coded transcripts and subsequently met to discuss coding and reach consensus [11]. Emergent themes were identified iteratively through constant comparison and team discussion. In the final analysis step, we mapped findings to i-PARIHS framework constructs.

## Results

The 22 PCPs interviewed ranged in age and clinical practice experience and reflected diversity in demographic characteristics, provider type, and practice setting (Table 1).

**Table 1:** Characteristics of Primary Care Providers (*N* = 22)

Characteristic	*N* (%)	Mean (SD)
Sex		
Male	11 (50)	
Female	11 (50)	
Provider Type		
MD/DO	15 (68)	
PA	3 (14)	
NP	4 (18)	
Practice Setting		
Hospital	12 (55)	
Community Clinic	10 (45)	
Geography		
Urban	17 (77)	
Rural	5 (23)	
Age		51 (11)
Years in Practice		14 (13)
Full-Time Equivalent		90 (20)
Panel Size		842 (271)

We identified three themes related to primary care provider HCV testing and linkage practices, as mapped to i-PARIHS constructs: 1) evaluating cues to HCV testing (innovation/evidence), 2) framing HCV testing decisions (recipients), and 3) HCV testing and linkage to care in the new treatment era (context). These themes are presented in Table 2 and in further detail below.

**Table 2:** Themes and Select Primary Care Provider Quotations

i-PARIHS Construct, Theme and Primary Care Provider Quotations	
Innovation: Evaluating Cues to HCV Testing	
• “I test everybody for hep C…you can’t tell who has hep C, you know? You can’t tell so it’s kind of like universal precautions. It’s like you have to assume everybody could have it.”	
• “They don’t know what’s out there so they just put the reminder up there and we just have to do it. No one asks our opinion how it should be, you know?”	
• “You get more reminders and more alerts and more steps and more barriers to everything you want to do and yet no diminution in the number of patients you’re expected to see and the more stuff you get the nurses to do, the more alerts you get, and you don’t have any time for that. So, you know, it’s just, I like taking care of patients, but the whole, the totality of the crap just gets more onerous all the time. “	
Recipients: Framing HCV Testing Decisions	
• “Frankly I was a little surprised to be contacted about this because it seems, at least with the public health/preventive medicine background, this seems like a no-brainer that we should just do this, but I do not.”	
• “If the patient has no risk factor and if…they’re still offered then the testing and if they say ‘No, I’m all set’ I don’t push them but if someone has the risk factors definitely then, you know, I put it in a different way so that it’s okay for them to have the test done…we go with the patient, whether yes or no.”	
• “I want time set aside to deal with things, so I try if at all possible to get their labs before they come in for their visit so that it’s taken care of at that time...so if they’ve already had their labs and the reminder pops up after the fact, and it doesn’t always occur to me, check what reminders are due at the time that I’m ordering labs.”	
Context: HCV Testing and Linkage to Care in the New Treatment Era	
• “I would say for me I think screening is screening and regardless of whether a patient can tolerate or pursue treatment I think it’s important to know and beyond myself personally I can’t really say one way or the other. I don’t know if people are not screening because they know patients aren’t going to be fit for treatment or if that’s changed based on the new treatments.”	
• “In terms of treatment for hep C I was very impressed when I came to the VA. We got a lot more people through treatment in the VA system than I ever got done outside the system.”	
• “I feel like it comes and it finds us rather than we go looking for it so it’s more like I’m not looking for it and that’s why I don’t test for it very often, like routinely, and because I don’t treat it, it makes it even less in my brain to look for it.”	

### Evaluating cues to HCV testing

The i-PARIHS innovation construct considers elements of evidence, knowledge, clarity, and Roger’s Diffusion of Innovations Theory components (i.e., relative advantage, compatibility, complexity, trialability, and observability) [12]. Providers described cues to HCV testing via their views on and experiences with the birth cohort guidelines, and how guidelines were understood, processed, deliberated, absorbed, and applied into practice.

Many PCPs reported that guideline details were unfamiliar, including one provider remarking: “I couldn’t repeat them for you.” Of those who were aware of the guidelines, several considered birth cohort screening “oversimplified.” Some PCPs were unconvinced by the epidemiological appropriateness and the strength of evidence for birth cohort screening, and questioned the validity and credibility of the guidelines, describing them as “somebody’s opinion [that] has relatively little basis on good scientific evidence.”

While PCPs acknowledged the possibility that there might be relative advantage to knowing one’s status, some still felt it was “a waste of time and money,” due to what they perceived as a “low yield” of observable positive test results. PCPs stated they rarely found “new positives” because most Veterans on their panels had been previously tested or had already been diagnosed. One provider expressed concern about misapplied effort: “it’s not a great diagnostic return to be fishing about for hepatitis C.” For others it was a low complexity “no-brainer” task that did not require much deliberation before offering.

A vocal minority of PCPs was critical that the guideline development process was neither transparent nor widely disseminated. One PCP noted that the policy priority may not have been aligned with their preferences as frontline providers: “no one asks our opinion how it should be.” Another lamented not being made aware of the practice change because otherwise “it takes a long time for guidelines to trickle down to community practices.” Some PCPs stressed that their clinical experience in the VA was to “test everybody anyway.” This was reinforced by a provider, who had previously practiced in a non-VA setting and recalled the contrast to VA: “when I was in the private sector I didn’t routinely offer a screening to everybody...I was never taught to ask specifically about hepatitis C.” Additionally, some PCPs did not perceive the birth cohort guidelines as a departure from past VA guidance to screen Vietnam-era Veterans (those who served between 1961 and 1975).

The guideline change was accompanied by updates to the computerized alerts or clinical reminder (CR) in the electronic medical record, which ultimately served as the central testing cue. PCPs often stated that the CR changes were an unwelcome “surprise” because they were not explicitly taught or disseminated. One provider observed: “when they brought out the older folks single once in a lifetime screening I don’t even think leadership mentioned it. I think it just kind of popped up and I realized, ah, we are doing that.”

Despite wide complaints about general CR fatigue, approximately half of the PCPs did not find the HCV testing reminders (sequence of risk assessment followed by test offer) to be burdensome. As one provider who welcomed the HCV CR, and CRs in general, conceded, “I find it annoying…but to me they are a valuable tool.” Another provider reported, “I’m not sure that I would pursue [HCV testing] as reliably if it weren’t for the reminder.” While the CR facilitated testing, it was perceived as a “blunt tool” with somewhat faulty underlying logic and frequent mechanical errors. One provider noted feeling like “a zombie” and functioning “on autopilot” when attending to CRs in practice. PCPs blamed glitches for some patients being inappropriately missed or tested repeatedly.

At the same time, there was opaqueness on current performance and whether improvement was indeed needed. Although PCPs received annual performance reports on clinical reminder completion for other metrics, HCV testing rates were not routinely reported to enable them to prompt action. Providers did not perceive a performance gap because they believed their “numbers” to be high, yet some were curious “…to see what our actual numbers are compared to my impression of the actual numbers.”

### Framing HCV testing decisions

The i-PARIHS recipients construct combines an understanding of the actors involved and with ongoing engagement with how their knowledge, skills, motivations, values, and beliefs enable or impede implementation of the new guidelines. PCPs described mixed motivations to test and inconsistently applied shared decision-making.

PCPs framed HCV testing as useful from an individual perspective insofar as it is “better to know than not to know.” Most PCPs reported practicing medicine through a “public health perspective”—offering tests as a “universal precaution” because “you have to assume everybody could have it” and “people should…take appropriate steps in terms of transmission of disease.” This was offset by PCPs’ view of HCV testing as a non-urgent priority “health maintenance” task that could wait, especially if other, more pressing needs existed.

PCPs expressed a preference for “case by case” approaches to conducting HCV testing–i.e., individual-driven rather than universal testing consistent with the new guidelines. Despite age being the sole criteria of the updated testing guidelines, PCPs’ ingrained practices of risk-based testing often resulted in continued consideration of social and behavioral risks (e.g., substance use histories and tattoos) as primary factors in testing decisions. For this reason, PCPs commented that an exhaustive HCV risk screen might induce “stigma” and jeopardize the patient-provider relationship. For example, providers voiced concerns about patients, particularly new patients, “lying,” or “failing to recall,” but in any case, omitting risk behaviors. Some PCPs explained that only after several visits and concerted effort to build rapport would patients become more forthcoming and “fess it up.” Alluding to recent trends in increased opioid use, one provider stressed the concern of substance use issues among Veterans: “I’m always suspicious of all of my patients for injection drug use.” Conversely, when considering older patients (even if in the birth cohort) providers “usually don’t bother asking [patients] at that point if they’re that elderly or frail.”

PCPs conveyed uncertainty over whether oral or written consent was required for HCV testing. Among those who knew the policy, many felt constrained by verbal consent requirement. PCPs tended to solicit verbal consent and believed it was “proper decorum to ask” if testing could be done rather than independently ordering a test. Many PCPs reported patient apprehension about testing, noting that “more decline than accept” when offered. While some PCPs believed it was worthwhile to “push” or emphasize the benefits of testing, others readily acquiesced if an offer was refused. Echoing the sentiment of many, one physician reported that “if the patient doesn’t want to get tested, that’s the only barrier I see.” Some PCPs were informal about getting permission and regarded the offer as “not really a discussion; it’s more of an informational statement,” thus offering little opportunity for patients to decline. In a minority of instances, PCPs ordered tests without explicitly informing the patient, sometimes as part of a pre-visit blood work panel.

Access to laboratory testing services was identified as a facilitator by many providers. However, providers at smaller community clinics did not have on-site phlebotomy, which prevented immediate access to blood draws and complicated pre-visit planning, given it is common practice to draw blood in advance of clinical visits. For established patients, if a test was not included in labs drawn prior to a clinic visit, PCPs reported waiting until the next visit to order the HCV test. Hence, the test might be forgotten or linger unresolved visit after visit, because as one provider remarked, “we follow as best we can…we can talk again in six months.” Alternatively, PCPs at locations with on-site laboratory services had fewer considerations about patient convenience regarding multiple blood draws. Moreover, ordering HCV tests in the EMR was perceived as not efficient: “It’s not on our order set. If I go to add new orders and I go outpatient labs, there’s all sorts of options, right?…Hepatitis C is not on here so I have to go to a special screen to do it…it doesn’t pop out in front of your face.”

### HCV testing and linkage to Care in the new Treatment era

Context, via i-PARIHS, exists at the local, organizational and external health system levels and together they represent leadership support and priorities, culture, structures and systems, and environment (in)stability. The single greatest context determinant was the newly encouraging HCV treatment landscape.

Some providers acknowledged that the lack of effective treatment options had previously been a deterrent to testing, but that recent “optimism” for curative treatments had led them to be more willing to offer tests. The rapidly improving treatment landscape was a source of testing encouragement. Nearly all PCPs were aware of and “excited” about the new treatments despite not having first-hand experience prescribing the “wonder drugs.” PCPs resolved to make new treatments available to their patients because, as one provider put it, “better test them early and get them started on some treatment to try to eradicate that.”

Most PCPs perceived HCV as a “specialist disease,” yet were familiar with the process of sending patients through the sequence of steps for treatment, often termed “the care continuum,” which involved diagnosis, linkage, retention and treatment. Providers at hospitals with co-located specialty care, compared to PCPs practicing in community clinics, expressed stronger relationships with HCV specialists. Nevertheless, most PCPs acknowledged that they were “confused because of the rapid succession of treatment protocols that have come out in the last couple of years.”

Upon notification of a positive result, PCPs sought to assuage Veterans’ fears of treatment side effects and encouraged them to attend follow-up specialty care; although many Veterans were “closed-minded” and patient follow-through created a barrier to referral completion and treatment initiation. Several PCPs reported linking patients to specialty care regardless of perceived treatment eligibility. Conversely, some PCPs were reluctant to link patients with active alcohol or substance use disorders as PCPs were cautious not to “waste [specialist] time and skill” if treatment could not be initiated due to active substance use. Several PCPs noted that an additional benefit of testing and linkage to treatment was that it served as a “big motivator to stay clean from drugs or alcohol.” Linking to specialty care was generally perceived as straightforward in operation. PCPs were mindful of having patients “packaged up for GI,” yet emphasized that there was no uniform methodology on how to “work up” patients prior to an HCV specialist visit.

## Discussion

To the best of our knowledge, this is the first study to examine primary care provider perspectives regarding HCV birth cohort testing in the new interferon-free treatment era in the VA. Our study identified multiple factors that influenced providers’ decisions to apply new birth cohort testing guidelines. We used the i-PARIHS framework to guide analysis, finding barriers and facilitators in the interaction of i-PARIHS’ innovation, recipients and context constructs. Absence of the fourth and unifying construct—facilitation—served as the key barrier to uniform and consistent uptake. Our findings help guide implementation strategy selection for quality improvement.

Where early guideline dissemination implementation strategies were somewhat unidimensional with a principal focus on knowledge and awareness, emphasis of multi-modal and multi-level interventions is emerging. Michie et al. identified that implementing new guidelines requires a combination of education, persuasion, incentivization, coercion, training, restriction, environmental restructuring, and enablement [13]. In our study, PCPs were often keen to test, diagnose, and link patients to specialists, but required support in several of these areas to do so. We describe considerations for quality improvement planning, including a number of which VA has made rapid progress on in recent years.

### Involve facilitators/knowledge translation experts in guideline dissemination

The updated guidelines were not perceived as a substantial departure and many felt they did not in fact change the way in which they were already practicing [14, 15]. Many also indicated that the VA missed an opportunity to raise PCPs’ awareness when the new guidelines were initially published. Failure to assimilate updated testing guidelines into practice may stem from PCPs’ reliance on longstanding, but outdated exclusively risk-based testing heuristics. Further, not all PCPs trusted the evidence that led to the birth cohort testing recommendations. Active guideline dissemination and information on subsequent changes to EMRs, lab procedures, etc. should be shepherded using champions, opinion leaders local to sites (internal facilitators) and knowledge translation experts (external facilitators) using multiple communication channels. Furthermore, these efforts should be jointly undertaken through experiential learning to ensure provider buy-in in the evidence-based practice and in the resulting process and/or behavior changes [7]. Since 2015, VA has operated the national Hepatitis C Innovation Team (HIT) Collaborative which led creation of regional quality improvement teams and provided a forum for the exchange of strong practices and system redesign improvement methods [16]. In addition, the VA maintains and updates guidance on hepatitis screening and management on an easily accessible public website, and appoints a Hepatitis Lead Clinician at each VA facility to be the principal point of contact for dissemination of viral hepatitis program information [17].

### Maximize utility of clinical reminders and decision support tools

The HCV CR, embedded in the electronic medical record, was often perceived as a helpful tool amidst general dissatisfaction with the abundance of CRs. Previous research has found that 33–96% of reminders are ignored, but that assigning reminder prioritization based on time-urgency and other relevant factors may increase provider compliance by 30–50% [18]. Once a classification system for reminders is established, better communication and coordination between policy, informatics and frontline users will serve as a low effort, high impact strategy to improve consistent CR use. To address redundancy issues, a revision to the national HCV testing CR was reprogrammed to skip behavioral risk questions in patients whose birth dates aligned with the birth cohort. Other healthcare systems have successfully improved HCV screening through embedding clinical reminders in EHRs [20, 21].

### Use audit and feedback to identify performance gaps

CR completion rate metrics lend themselves well to informing performance reports; however, this opportunity had not been taken advantage of at the time of interviews. In the absence of performance data, PCPs in this study almost always believed that their HCV testing performance was excellent and that the majority of their panels had already been tested. Such an “overconfidence effect” has been shown to limit the ability to identify and correct mistakes [22]. To correct for the dearth of data, audit and feedback strategies with appropriate benchmarking could be implemented to help identify performance gaps and direct improvement efforts [23, 24]. Notably, the VA made testing a national and regional VA quality performance metric and since 2016 has been operating a national HCV dashboard for real-time reporting on testing and treatment metrics [2, 17].

### Train, educate, and support providers

Our results suggest that concerns, and in some cases inaccurate understanding, about consent requirements were barriers to testing. Following this work and widespread concerns form other providers the VA updated its consent policy to no longer require documentation of verbal consent in the patient’s EMR [24]. However, practical and ethical questions remain as to how consent should be presented and whether these discussions should be qualitatively different in nature than discussions about other laboratory tests (e.g. cholesterol).

The availability of new, easy to take, and curative antiviral medications intensified the importance of HCV identification and served as the main facilitator to testing [4]. While there were mixed perspectives on the utility of HCV testing, PCPs unanimously valued treatment. For providers the evidence of efficacious treatment offset the risk of a potential new HCV diagnosis; however, providers reported that patients remained reluctant to pursue treatment. Providers could benefit from updating approaches to testing and linkage to care conversations in order to allay patient reticence to be tested and linked to curative treatments. The role of primary care in HCV is further expanding: a result of simpler and more efficacious HCV treatment regimens, studies are demonstrating HCV is now amenable to treatment by non-specialists in primary care settings [25, 26].

Testing rates among Veterans enrolled in VA care are substantially higher than community primary care settings [27]. VA has steadily improved Baby Boomer HCV testing performance. Prior to VA testing guideline updates (end of 2012), 64% of Baby Boomers in VA care had been HCV tested. By 2015, 70% of Baby Boomers in VA care had been HCV tested, and by September 2018, 84% had been tested. In contrast, by 2015 only 14% of non-VA Baby Boomers in community settings had been tested [29]. Yet, only about half of US Veterans are currently enrolled in and receiving VA care, thus leaving many with undiagnosed HCV in the care of non-VA community providers or not in regular care [28]. As a result, more than half of those living with HCV are unaware of their status [29].

#### Strengths and limitations

A strength of this study is the timing of interviews coincided with the diffusion of two new practices: birth cohort testing and interferon-free treatment. The qualitative approach enabled an in-depth exploration of implementation issues, however recall and desirability bias are limitations, as with other qualitative work. The findings may be transferrable to care in many safety-net care settings, especially those, like New England, that serve vulnerable populations with significant opioid epidemics and large concentration of Baby Boomers; however, the study was conducted in one region and findings may not be representative of other healthcare systems, many of which are not integrated and care for larger female patient populations.

## Conclusions

Using an implementation science framework that focuses on the tetrad of innovation, recipients, context and facilitation to analyze our data, we identified key features underlying practice decisions to test for HCV and link to specialty care for treatment. While many PCPs were unaware of details of the birth cohort testing guidelines and had not integrated them into practice, they were motivated to test and receptive to clinical reminders and other cues to test. These findings suggest that a multi-component intervention including efforts on awareness and education, feedback of performance data, clinical reminder updates, and leadership support, may be both accepted and feasible routes of intervention to improve HCV testing in VA.

## Additional file

Additional file 1: Qualitative Interview Guide. These are the questions asked during individual qualitative interviews with primary care providers. (DOCX 15 kb)

## Abbreviations

CR: Clinical reminder
EMR: Electronic medical record
GI: Gastrointestinal
HCV: Hepatitis C virus
i-PARIHS: Integrated Promoting Action on Research Implementation in Health Services
PCP: Primary care provider
US: United States
VA: Department of Veterans Affairs
