# Supplementary material for: Primary care provider perceptions and experiences of implementing hepatitis C virus birth cohort testing: a qualitative formative evaluation
Source: BMC Health Serv Res. 2019 Apr 23;19:236. doi: 10.1186/s12913-019-4043-z (PMC6480846; doi:10.1186/s12913-019-4043-z)
Supplement: Supplementary file 1 — Qualitative Interview Guide. These are the questions asked during individual qualitative interviews with primary care providers. (DOCX 15 kb) [file 12913_2019_4043_MOESM1_ESM.docx]

**Additional file 1. Interview Guide**

**Recipients**

1. How long have you been a primary care provider in the VA? Do you practice outside of the VA?
2. Please tell me about your primary care practice [number of clinics per week, panel size].
3. How many or estimate how many of your patients have HCV?
4. How do you determine who is a candidate for HCV testing? What influences your decisions to offer HCV testing? How do you decide whether or not to offer an HCV test? Can you give me an example of when you decided to not offer a test?
5. Do you typically assess for HCV risk in your patients? What factors do you consider when assessing risk for HCV? What kinds of conversations are you having with Veterans about HCV testing?

**Evidence & Innovation**

1. What role do HCV testing guidelines or recommendations play in your offer of an HCV test to your patients? The VA has recently changed their guidelines to indicate that all patients born between 1945 and 1965 should be tested for HCV. What do you think of this birth cohort based strategy, as opposed to the risk-based strategy that the VA currently uses for all Veterans?
2. How will the testing guidelines impact your practice? How do you expect the VA change in guidelines will impact how you managing testing for this group? How hard will this be to do? Are there resources that you will need in order to implement these guidelines?
3. How do you currently manage risk assessment for patients who are not born between 1945 and 1965?

**Context**

1. If you practice outside of the VA, how does your other practice differ from VA with regard to HCV testing?
2. How much of a priority is HCV testing in relation to other clinical tasks?
3. What is the level of VA, facility and local leadership support for HCV testing?
4. What works well at your facility for HCV testing? Which of these facilitators is most influential?
5. What do you find challenging about testing or expanding testing for HCV? What gets in the way [barriers] of testing?

**Facilitation**

1. Are there things that would help in order to improve HCV testing at your facility? [policies, leadership, logistical, buy-in]. In an ideal world, what changes would you make in order to improve testing?
